# The synergistic use of plant and isolated bacteria to clean up polycyclic aromatic hydrocarbons from contaminated soil

**DOI:** 10.1186/s40201-017-0274-2

**Published:** 2017-06-17

**Authors:** S. Eskandary, A. Tahmourespour, M. Hoodaji, A. Abdollahi

**Affiliations:** Isfahan(Khorasgan) Branch, Islamic Azad University, Arghavanieh Bv, Jey street, Isfahan, Iran

**Keywords:** Soil Pollution, Phytoremediation, Rhizosphere, Bacteria, PAH

## Abstract

**Background:**

Biological methods of polycyclic aromatic hydrocarbons (PAH) contamination elimination typically involve the transformation of contaminants to non-toxic materials by microorganisms and plants and appear to be the most effective methods available.

**Methods:**

In this study, *Bacillus licheniformis* and *Bacillus mojavensis* isolated from oil-contaminated soils were inoculated onto *Festuca arundinacea* seeds before planting in the pot and 3 weeks after planting by syringe injection into the rhizospheric zone in order to study the elimination of PAHs from *Festuca*’s rhizosphere in the greenhouse. Some physical and chemical properties of the soil, PAH concentrations, seeds germination percentage, root and shoot biomasses of the treated samples were examined.

**Results:**

The results showed that the treated samples inoculated with both bacteria had a significantly higher percentage of seed germination and root and shoot biomass compared to other treatments. The concentration of some PAHs reduced significantly (Pvalue < 0.05) in the rhizosphere of the treated samples inoculated with both bacteria compared to in contaminated soils. Concentrations of some PAHs (eg. Naphthalene, Phenanthrene, Benzo[a]anthracene and Dibenzo[a,h]anthracene) even reached below the detection limit of the method. The PAHs concentrations in the treated samples inoculated with bacteria was decreased significantly (Pvalue < 0.05). Therefore, the results showed the high efficiency of the Festuca and bacterial inoculation in eliminating PAHs from the soil.

**Conclusion:**

According to the results, the partnership of *Festuca* with *B. licheniformis* and *B. mojavensis* isolates displayed positive effect on PAHs dissipation and can be effective cleanup technology with high performance.

## Background

PAHs are aromatic compounds with two or more benzene rings. They are usually produced by the thermal decomposition of organic molecules and similar compounds [1]. The common sources of these compounds in the environment might be human activities, wildfire, oil spills and volcanoes. These compounds are known as toxic, mutagenic and carcinogenic pollutants [2] and can not easily eliminate from the environment under natural conditions; and as their molecular weight increases, their resistance to degradation also increases. Due to their widespread presence in the air, soil and sediments, they have attracted a lot of attentions in different studies. These compounds should be eliminated from the environment because evidences indicate that PAHs mixtures are teratogenic, mutagenic and carcinogenic to humans [3]. Different methods such as biological, thermal, physical, chemical and solidification can be applied for soil remediation [4]. Among the different methods of remediation, biological techniques are often regarded as cost – effective and environmental friendly [5].

Bioremediation is the productive utilization of living systems to degrade, detoxify, transform, immobilize or stabilize toxic environmental contaminants which require the least amount of energy and chemical substance. One of the bioremediation strategies is phytoremediation which is the use of plants for contaminant removal from the soil. Phytoremediation makes use of advantages such as the plants’ complex root system, which occupies a large body of the soil. The root system also protects a large population of bacteria in the rhizosphere and produces secretions that can directly impact the activity of the bacterial population present in the rhizosphere and eliminate contamination. Plants used for this purpose should have more flexibility and compatibility in stress conditions caused by the presence of hydrocarbons [6]. The symbiotic relationship between plants, microorganisms and the environment is one of the new methods for cleaning oil-contaminated soils. So, it is believed that isolation of indigenous bacteria with the ability to degrade PAHs and their addition to the rhizosphere of plants (such as Festuca) can improve the remediation efficacy. Extensive attention has been paid on PAHs degradation by gram-negative bacteria, but, less attention has been intended on the PAHs degradation by gram-positive bacteria of *Bacillus* species [7] and also their partnership in the rhizosphere of plants as such Festuca.

Doyle [8] has shown that a higher level of PAH degradation occurs close to the plant roots [8]. A study conducted in 2013 by Liu et al. on the effects of oat on the phenanthrene degradation in soil showed that growing oat in soils contaminated with this compound stimulates its degradation. As a result, the concentration of phenanthrene in the cultivated soil after harvest was significantly reduced compared to in non-cultivated soil (*P* < 0.05). They also stated that oat cultivation stimulates microbial biomass first and ultimately phenanthrene degradation [9]. Although abiotic factors such as pH, moisture, oxygen and available nutrients can affect PAH degradation, microbial processes have a notably greater share in the degradation of these compounds [10]. Specific populations of decomposing bacteria are absorbed to petroleum hydrocarbons. Also, there is plant-microbe interaction in bioremediation process. However, plants, throughout their ‘rhizosphere effects’, can support hydrocarbon-degrading bacteria that assist in bioremediation in the root zone [1]. Application of plants in combination with some microorganisms to increase the efficiency of contaminants bioremediation can be valuable [11]. Banks et al. [6] stated that when herbaceous plants are infected with bacteria, they can decompose a wide range of chlorobenzoic acids. A wide range of herbaceous plants and legumes, along with their symbiotic bacteria, have shown to increasingly remove oil contaminations from the soil [6].

Therefore, we hypothesized plants inoculated with indigenous PAHs degrading bacteria may enhance bioremediation efficiency of petroleum contaminated soils in comparison to non-inoculated plants. The objectives of this study were to isolate PAHs degrading bacteria from polluted soil and investigate the effect of *Festuca arundinacea* inoculated with and without indigenous isolated bacteria (*Bacillus licheniformis* and *Bacillus mojavensis*) to clean up PAHs from contaminated soil.

The Festuca was selected due to its relatively fast growth, and that is a natural plant of Iran. Moreover, *F. arundinacea* has been used in several phytoremediation studies on oil contaminated soils without inoculated with such isolated [6, 12].

## Methods

### Sampling

The soil around refinery tanks of oil and gas, which often exposed to contamination by oil and its derivatives, was randomly selected for this study. With the aid of hand trowel, soil samples were collected in plastic bags from ranges 0–30 cm of the contaminated soil around Esfahan oil and gas refinery in Esfahan province, Iran (32°47′19˝N, 51°30′24˝E) and mixed together. The samples (~50 Kg) were then air dried, grounded and passed through 2 mm sieve and immediately transferred to the laboratory. The soil was used for isolation of PAHs-degrading bacteria (10 gr) and also for bioremediation experiments.

### Measurement of physical and chemical properties of the soil

After transferring the soil samples, their pH, electrical conductivity, and organic matter were measured. The soil pH was measured in its saturated extract using a pH meter (model 262, Horiba) calibrated with buffer solutions [13]. The Walkley-Black method was used to measure the soil organic matter (OM). Type of soil texture was defined using a hydrometer and the electrical conductivity (EC) of the saturated extract was determined using a conductivity meter [14].

### Screening and identification of PAHs-degrading bacteria

PAHs-degrading bacteria were isolated from contaminated soils using an enrichment culture technique in Basal Salt Medium (BSM) supplemented with 12.8 mg/L PAHs as sole sources of carbon and energy. The BSM contained per liter: KH_2_PO_4_ of 1.0 g, Na_2_HPO_4_.12H_2_O of 1.25 g, (NH_4_)_2_SO_4_ of 1.0 g, MgSO_4_.7H_2_O of 0.5 g, CaCl_2_.6H_2_O of 0.05 g, FeSO_4_.7H_2_O of 0.005 g, and pH of 7.0 (pH meter, Metrohm 827). The medium was autoclaved at 121 °C for 20 min. The PAHs mixture (sigma Aldrich Co.) was filter-sterilized (using 0.22 μm pore film) and added at final concentrations of 12.8 mg/L to the Erlenmeyer. The solvent was allowed to evaporate on a rotary shaker before adding the BSM and soil sample. The amount of 50 ml sterilized BSM was added to the Erlenmeyer flask, and 1 g of contaminated soil was suspended in it*.* All cultures and controls (without PAHs) were incubated for 7 days at 30 °C on a dark rotary shaker (Vision 8480 SFN) at 150 rpm. At the end of each week, 10% of the cultured medium was transferred to the fresh medium. After five consecutive enrichments, 0.5 ml of the last enrichment cultures were spread onto solidified BSM plates (2% agar) and sprayed with concentrated PAH solutions. The plates were then incubated at 30 °C and routinely checked for colony growth. Colonies with different morphology were picked and streaked onto fresh nutrient agar plates to obtain pure culture [15, 16].

Identification of isolates was performed by morphological and biochemical characteristics [17]. Further identification of isolates was done using the 16S rDNA gene amplification by PCR (Thermocycler, Eppendorf 632500) using universal primers 27 F (5′-AGAGTTTGATCCTGGCTCAG-3′) and 1492R (5′-GGTTACCTTGTTACGACTT-3′). Two isolates, *Bacillus licheniformis* ATHE9 and *Bacillus mojavensis* ATHE13 with NCBI accession numbers of *KC329470* and KC469987 were finally selected and used for seeds inoculation [18].

### Inoculation of bacteria into seeds of Festuca arundinacea

The seeds were first surface sterilized by soaking in 70% (v/v) ethanol for 1 min and rinsed 3 times with sterile distilled water and then they were soaked in 1:10 (v/v) dilution of commercial hypochlorite bleach for 15 min and rinsed several times with sterilized distilled water [19]. For inocula preparation, the isolated bacteria were grown overnight. Cells were harvested in exponential phase (OD600 ~ 0.8) and centrifuged at 3000 rpm for 10 min. The first inoculation of seeds with bacterial cells was done before planting. For this, sterilized seeds were soaked in bacterial cell suspension (cell density 9 × 10^8^ CFU/ml) for 30 min. Then; they were planted (without rinsing) in the plastic pots 27 cm in height and 25 cm in diameter containing 3Kg of polluted soil. Further inoculation (1 ml with the same concentration) was done three weeks after planting by syringe injection into the rhizospheric soil (For control treatment the same volume of water was injected into the soil). The seeds without bacterial inoculation were used as the control treatment [20, 21].

### Bioremediation of contaminated soil

The above plants were cultivated under controlled conditions in a greenhouse for a period of 3 months, during which time they were exposed to sun light for 18 h daily with a maximum temperature of 30 °C and a minimum temperature of 20 °C. All the pots were watered based on 75% of soil field capacity. There was no drainage available that showed no water leaching out of the pots. After that their growth period was completed, the soil around the roots of each treated sample was gently and carefully separated and then transferred to the laboratory for extracting purposes [22]. There were three replicates in each treatment.

### Measurement of PAH concentrations

The present study examined 10 polycyclic aromatic hydrocarbons, that is, Naphthalene, Acenaphthene, Acenaphthylene, Phenanthrene, Chrysene, Anthracene, Benzo[a]anthracene, Benzo[a]pyrene, Dibenzo[a,h]anthracene, Benzo[ghi]perylene. To examine PAH concentrations, 5 g of soil sample passed through 2 mm sieve with 5 g of activated sodium sulfate and a mixture of 150 ml of acetone and dichloromethane with a 1:1 volume ratio and was then extracted using a Soxhlet extractor. After reducing the soil sample volume through a rotary evaporator and drying it with nitrogen gas, it was kept in the freezer at −20 °C until injection. At the time of the injection of soil extracts to the Agilent 7890a GC-FID, specific concentrations of an internal standard with m-Terphenyl were added to all the samples [23, 24]. An HP5 column with an internal diameter of 250 micrometers, a length of 30 meters and a thickness of 0.25 micrometers was used to measure the PAHs with the GC-FID. The GC-FID was programmed with an inlet temperature of 260 °C and a nitrogen carrier gas flow of 1.5 ml per minute. The device’s initial temperature started at 70 °C and reached 290 °C with a gradient of 5 °C, and then reached 305 °C with a gradient of 1 °C per minute. Detector temperature was taken as 270 °C in this program. Field and laboratory blanks were extracted with each batch of sample to reflect laboratory contamination variability. For a compound to be positive, the sample must have exceeded the method detection limit (MDL), defined as MDL = mean blank + 3 × SD of the blank. Where there were no peaks in the blanks, the instrumental detection limits (IDLs) were used. The IDL defined as the amount of analyte that generated a signal to noise ratio of three.

### Statistical analysis

The experiment was carried out in a randomized complete block design with three replications. The treatments were designed in the form of *Festuca arundinacea* inoculated with *B. licheniformis* ATHE9 (F_1_), *Festuca* inoculated with *B. mojavensis* ATHE13 (F_8_), *Festuca* inoculated with both bacteria (F_1,8_) and *Festuca* without inoculations (F_0_). Statistical analysis of the experimental data was performed using statistical software known as SAS (Release 9.1). Mean comparisons were done with the Duncan test at a significance level of 0.05.

## Result and Discussion

### Soil properties

Some physical and chemical properties of the soil were measured, which are presented in Table 1. The availability of nutrients and even contaminants depends on the degree of soil acidity. The most suitable pH for soil microorganism’ activity is 7, as nutrient solubility depends on soil pH changes [25]. The soil used in the present study has neutral pH and therefore does not limit the activity of microorganisms or the solubility of nutrients except for phosphorus. According to the results provided in Table 1 and using the soil texture triangle, the type of soil was identified as loamy sand. Since the clay content percentage of this soil is low, the adsorption of hydrocarbons to these particles is also decreased and they become more prone to microbial degradation. Afzal et al. [26] suggest that soil type affects the bacterial colonization and microbial activities and thus the efficiency of contaminant degradation. The highest levels of hydrocarbon degradation were seen in loamy soil in their study [26]. The appropriate content of organic matter in the soil is approximately 5%. In the examined soil, this content was 4.6%, pertaining only to contaminants, as the examined soil lacked vegetation for a long time. The cation exchange capacity is 7 (meq/100 g^−1^), which is rather low, and might be attributed to the low clay content of the soil. Lease [27] mentioned that soils physicochemical properties such as organic matter affect organism’s ability to establish and achieve degradation in the soil environment significantly [27].

**Table 1:** Physical and chemical properties of the soil before land remediation

pH	EC (dS m^−1^)	CEC (meq 100 g^−1^)	OM (%)	Clay (%)	Sand (%)	Silt (%)	FC water content %)
7.24 ± 0.04	3.4 ± 0.25	7 ± 1	4.6	16.6	75	8.4	22.4

*EC* Electrical Conductivity, *CEC* Cation Exchange Capacity, *OM* Organic Matter, *FC* Field Capacity

### The PAHs concentration in contaminated soil

The concentrations of PAHs in the soil treatments are listed in Table 2. Results demonstrate that the concentration of these hydrocarbons in soil is much higher than the acceptable range of 50 to 1100 μg/kg [28]. These results are indicative of the high level of contamination in the soil, confirming the necessity of cleansing the soil from hydrocarbons.

**Table 2:** Concentration of Heavy (H) and Light (L) polycyclic aromatic hydrocarbons in the soil treatments

	Hydrocarbon	Concentration (mg kg^−1^)				
		Untreated soil	Treated soil			
			F1	F8	F1,8	F0
L PAHs (2–3 rings)	Naphthalene	16 ± 6	2.4 ± 0.3^b^	ND^c^	ND^c^	12.2 ± 0.9^a^
	Acenaphthene	18 ± 4	2.9 ± 0.14^b^	1 ± 0.2^c^	3.5 ± 0.7^b^	5.7 ± 1.5^a^
	Acenaphthylene	32 ± 9	4.7 ± 1^b^	2.2 ± 0.14^b^	2.6 ± 0.5^b^	5.7 ± 2^a^
	Anthracene	4.5 ± 2	1.5 ± 0.3^b^	ND^b^	1.3 ± 0.12^b^	4 ± 1.2^a^
	Phenanthrene	6 ± 2	1.5 ± 0.4^b^	ND^b^	ND^b^	3.2 ± 1.25^a^
H PAHs (4–6 rings)	Benzo[a]anthracene	22 ± 8	2.3 ± 0.3^b^	ND^c^	ND^c^	17 ± 3.19^a^
	Benzo[a]pyrene	5 ± 1.3	2.6 ± 0.28^ab^	1.7 ± 0.4^b^	2.2 ± 0.5^ab^	3.6 ± 0.55^a^
	Benzo[ghi]perylene	2.5 ± 1	ND^b^	2.2 ± 0.14^a^	1.6 ± 0.4^a^	2.4 ± 0.15^a^
	Chrysene	16 ± 6	3.4 ± 0.24^c^	5.1 ± 0.35^b^	ND^d^	16 ± 3.65^a^
	Dibenzo[a,h]anthracene	8 ± 2	3.9 ± 0.28^b^	3.9 ± 0.2^b^	ND^c^	6 ± 1^a^

*F1: Festuca* with *B. licheniformis* ATHE9, F8: *Festuca* with *B. mojavensis* ATHE13, F1,8: *Festuca* with both bacteria (F1,8) and F0: *Festuca* without inoculations (control), Means with different letters (a, b and c) are significantly different at 5% level test. ND: Not detected (below the detection limit (DL) of method), DL = 0.03 ± 0.015 mg kg^−1^

### Some physiological properties of the plant

**Fig. 1:**
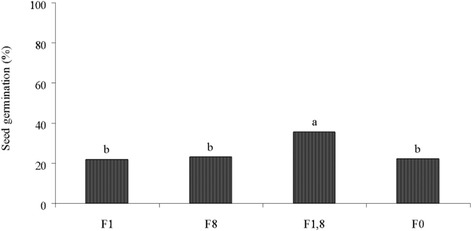
The percentage of seed germination in the treatments. *F1: Festuca* with *B. licheniformis* ATHE*9*, F8: *Festuca* with *B. mojavensis* ATHE13, F1,8: *Festuca* with both bacteria (F1,8) and F0: *Festuca* without inoculations (control), Means with different letters (*a*, *b*) are significantly different at 5% level

**Fig. 2:**
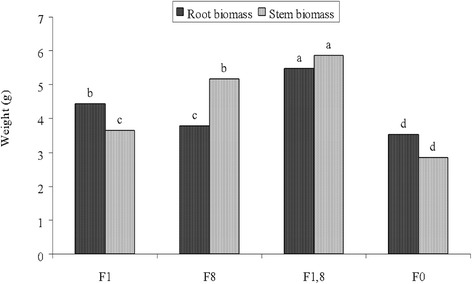
Root and shoot biomasses of the treated samples. *F1: Festuca* with *B. licheniformis* ATHE9, F8: *Festuca* with *B. mojavensis* ATHE13, F1,8: *Festuca* with both bacteria (F1,8) and F0: *Festuca* without inoculations (control), Means with different letters (*a*-*d*) are significantly different at 5% level

Some of the plant properties such as seed germination percentage, root, and shoot biomass of the treated samples are shown in Figs. 1 and 2. The germination percentage of Festuca in contaminated soil has severely decreased in the treated samples from approximately 85% in non-contaminated soil (data not shown in Fig. 1) to about 20% in contaminated soil. As shown in Fig. 1, the treated sample inoculated with both bacteria (F_1,8_) had a higher germination percentage with a significant difference (p < 0.05) compared to other treated samples, thus improved germination conditions in the presence of both bacteria is indicated. It is possible that the presence of hydrocarbons in soil can affect the ability of the soil to provide water and oxygen for the seeds, thus delaying the germination in the presence of hydrocarbons or other toxicities are may be due to preventing water and oxygen infiltrating to the seeds [29]. Increasing the seeds’ germination percentage of inoculated plant with both bacteria (F_1_,_8_) can reflect that, maybe a good amount of water and oxygen infiltrates the seeds and therefore improves their germination percentage. The inoculated bacteria belonged to the Bacillus species which are studied extensively for the production of secondary metabolites and surface active compounds, such as surfactin, fengycin, lichenysin, iturin, pumilacidin and bacillomycin. Biosurfactant helps to solubilize or mediate the interaction between the bacterium and the compound [7]. So, *Bacillus* species are the good choice for bioremediation studies. Jalilzadeh Yengejeh et al. also showed that *B. subtilis* has the ability of oil hydrocarbons biodegradation and biosurfactant production [30]. Shahidi and colleagues also indicated that Bacillus species can biodegrade crude oil and similar compounds [31].

In the majority of the research conducted on herbaceous plants, rhizospheres are used for the degradation of hydrocarbons. Degradation is facilitated through the effect of rhizosphere; roots of plants secrete organic compounds, increasing the density and activity of potential hydrocarbon-degrading bacteria in the area around the roots. Herbaceous plants are therefore chosen for phytoremediation due to their diffuse root system, extensive capillary roots and higher amounts of enzyme production [32].

Festuca plant seeds are more sensitive to contaminated conditions compared to other plant seeds. In a similar study, the germination of Tall Festuca, Red Festuca and Perennial rye-grass was studied in PAH-contaminated soil with a concentration of 5000 mg/kg. The best germination was reported by rye-grass with 2 days of delay while the low germination rate pertained to Red Festuca with 9 of days’ delay [33]. Figure 2 presents the root and shoots biomass of *Festuca arundinacea* plant after treatments. As evident by the figure, the root and shoot biomasses were significantly higher in the plant inoculated with both bacteria (p < 0.05) compared to other treatments. One research has shown that, in contaminated conditions, root and shoot biomasses also decrease as the plant wastes most of its energy adjusting to the contaminated conditions and absorbing water and nutrients from the soil [34]. The results of this study indicate that the inoculation of both bacteria has balanced the hydrocarbon-contaminated conditions of the soil for the plant to some degree in order for the plant to invest more energy into producing biomass.

### The PAHs concentration of treated soils

The concentration of PAHs, 2–3rings (Light) and 4–6 rings (Heavy), in soil under different treatments after 3 months of bioremediation (rhizoremediation) are presented in Table 2. According to the results, there were some decreases in the PAHs concentration of control treatment (F0) in comparing with untreated soil that is related to the effect of Festuca by means of phytoremediation. The concentrations of hydrocarbons in the rhizosphere of treated soils significantly (p < 0.05) decreased compared with the initial soil PAH concentrations and also in comparison with F0 significantly. In treatment F_1,8_, the residual levels of benzo[a]anthracene, benzo[ghi]perylene, chrysene and dibenzo[a,h]anthracene were lower than other treatments (more than 95% degradation). The reduction of such PAHs concentration in the F_1,8_ treatment can be related to the synergistic effect of rhizobacteria (*B.mojavensis and B.licheniformis*) and plant in remediation of polluted soil which is known as rhizoremediation. Rhizoremediation application in crude oil contaminated soil has been confirmed as a proper and useful option to the cleanup of petroleum hydrocarbon in the contaminated environments [35]. In this synergistic process, bacteria are the main contributors to the degradation process. Plants release enzymes, amino acids, sugars and low molecular weight carbohydrates into the soil ecosystem that can increase the microbial activity and support the degradation of xenobiotic substances along with absorbing and accumulating or translocating the xenobiotics to the shoot, root or other parts of it [36]. Andria et al. [37] showed that plants inoculated with the endophyte were better able to grow in the presence of diesel. Higher expression of *alkB* genes suggesting a more efficient degradation of the pollutant was observed in their study [37].

**Fig. 3:**
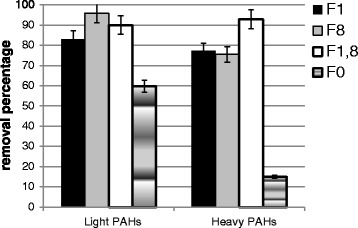
The removal percentage of polycyclic aromatic hydrocarbons in the treated soil. *F1: Festuca* with *B. licheniformis* ATHE9, F8: *Festuca* with *B. mojavensis* ATHE13, F1,8: *Festuca* with both bacteria (F1,8) and F0: *Festuca* without inoculations (control)

The concentration decrease of 2- and 3- ring hydrocarbons in the rhizosphere were higher than heavier hydrocarbons in all treatments (e.g. 95.8% vs. 75,9% in F_8_ treatment) (Fig. 3). Heavy hydrocarbons were less exposed to microorganisms due to their lower solubility in an aqueous phase and higher adsorption on soil surfaces; as a result, their degradation is also lower. Haritash and Kaushik [10] stated that the presence of light oils instead heavy ones can increase the bioavailability of PAHs [10]. Soleimani et al. [12] reported the lowest degradation degree of benzo (ghi) perylene (a 6-ring PAH) and concluded that high-molecular-weight PAHs do not easily serve as a carbon and energy source for microbial populations during degradation. They also stated that benzo (ghi) perylene could be removed in the presence of plant root exudates through the co-metabolism mechanism [12].

Also, results showed that in treatments of *Festuca* inoculated with bacteria (F_1_, F_8_, F_1.8_) the removal percentages of PAHs were significantly (p < 0.05) more than *Festuca* without inoculation (F_0_). Given these findings, it can be asserted that *B. mojavensis* and *B. licheniformis* have the potential for improving the conditions for remediation. The different degrading behavior of bacteria toward the types of hydrocarbons (light or heavy) can be attributed to the unique properties of each bacterium. Each bacterium acts uniquely in its degradation of certain hydrocarbons based on its specific enzyme system [27]. According to results of the present study, it can be asserted that the mechanism of bio and phytoremediation (rhizoremediation), which involves the plant and its dependent microbial activities in the rhizosphere, is greatly capable of removing PAHs from the soil. In such plant–bacteria partnership, plant supplies the bacteria with a special carbon source that stimulates them to degrade organic contaminants in the soil. In response, bacteria can support the plant to conquer contaminated-induced stress responses, and improve plant growth and development. Additionally, plants get benefits from the bacteria with hydrocarbon-degradation ability, leading to enhanced hydrocarbon mineralization and lowering of both phytotoxicity and evapotranspiration of volatile hydrocarbons [38, 39] and improved the efficiency of phytoremediation [40]. Su and Zhu [22] also showed the relative contributions of plant uptake and plant-promoted rhizosphere microbial biodegradation of PAHs in a different environment [22].

## Conclusion

Rhizoremediation process is an appropriate method for eliminating PAHs from the soil. In the present study, *Festuca* was inoculated with isolates of *B. licheniformis* ATHE9 and *B. mojavensis* ATHE13 which displayed a positive effect on PAHs dissipation. This can be a smart solution to combine the advantages of microbe-plant symbiosis within the plant rhizosphere into an effective cleanup technology with high performance. This method may be a promising strategy to remediate more contaminated sites along with the sustainable production of non-food crops for biomass and biofuel production. Therefore, it may become attractive in developing countries because not expensive and requires little management.

## Abbreviations

BSM: Basal Salt Medium
CFU: Colony Forming Unit
EC: Electrical Conductivity
OM: Organic Matter
PAH: Polycyclic Aromatic Hydrocarbons
PCR: Polymerase Chain Reaction
